# The Value of In Vivo Reflectance Confocal Microscopy as an Assessment Tool in Chemotherapy-Induced Peripheral Neuropathy: A Pilot Study

**DOI:** 10.1093/oncolo/oyac106

**Published:** 2022-06-15

**Authors:** Sabrina R Ramnarine, Patrick M Dougherty, Roman Rolke, Linda J Williams, Christi Alessi-Fox, Andrew J Coleman, Caterina Longo, Lesley A Colvin, Marie T Fallon

**Affiliations:** Clinical Imaging and Medical Physics, Guys’ and St. Thomas’ NHS Foundation Trust, London, UK; Faculty of Life Sciences & Medicine, King’s College London, London, UK; Edinburgh Cancer Research UK Centre, MRC Institute of Genetics and Molecular Medicine, University of Edinburgh, Edinburgh, UK; Department of Pain Medicine, Division of Anesthesiology, Critical Care and Pain Medicine, The University of Texas MD Anderson Cancer Center, Houston, TX, USA; Department of Palliative Medicine, Medical Faculty RWTH Aachen University, Aachen, Germany; Edinburgh Clinical Trials Unit, Usher Institute, University of Edinburgh, Edinburgh, UK; Caliber Imaging and Diagnostics Inc., Rochester, NY, USA; Clinical Imaging and Medical Physics, Guys’ and St. Thomas’ NHS Foundation Trust, London, UK; Department of Dermatology, University of Modena and Reggio Emilia, Modena, Italy; Azienda Unità Sanitaria Locale-IRCCS di Reggio Emilia, Centro Oncologico ad Alta Tecnologia Diagnostica-Dermatologia, Reggio Emilia, Italy; Division of Population Health and Genomics, University of Dundee, Dundee, UK; Edinburgh Cancer Research UK Centre, MRC Institute of Genetics and Molecular Medicine, University of Edinburgh, Edinburgh, UK

**Keywords:** peripheral neuropathy, neurotoxic chemotherapy, in vivo reflectance confocal microscopy, Meissner’s corpuscles, Quantitative Sensory Testing, patient-reported outcome measures

## Abstract

**Background:**

There is a lack of standardized objective and reliable assessment tools for chemotherapy-induced peripheral neuropathy (CIPN). In vivo reflectance confocal microscopy (RCM) imaging offers a non-invasive method to identify peripheral neuropathy markers, namely Meissner’s corpuscles (MC). This study investigated the feasibility and value of RCM in CIPN.

**Patients and Methods:**

Reflectance confocal microscopy was performed on the fingertip to evaluate MC density in 45 healthy controls and 9 patients with cancer (prior, during, and post-chemotherapy). Quantification was completed by 2 reviewers (one blinded), with maximum MC count/3 × 3 mm image reported. Quantitative Sensory Testing (QST; thermal and mechanical detection thresholds), Grooved pegboard test, and patient-reported outcomes measures (PROMS) were conducted for comparison.

**Results:**

In controls (25 females, 20 males; 24-81 years), females exhibited greater mean MC density compared with males (49.9 ± 7.1 vs 30.9 ± 4.2 MC/3 × 3 mm; *P = .*03). Differences existed across age by decade (*P < .*0001). Meissner’s corpuscle density was correlated with mechanical detection (*ρ* = −0.51), warm detection (*ρ* = −0.47), cold pain (*ρ* = 0.49) thresholds (*P < .*01); and completion time on the Grooved pegboard test in both hands (*P* ≤ .02). At baseline, patients had reduced MC density vs age and gender-matched controls (*P = .*03). Longitudinal assessment of MC density revealed significant relationships with QST and PROMS. Inter-rater reliability of MC count showed an intraclass correlation of 0.96 (*P < .*0001).

**Conclusions:**

The findings support the clinical utility of RCM in CIPN as it provides meaningful markers of sensory nerve dysfunction. Novel, prospective assessment demonstrated the ability to detect subclinical deficits in patients at risk of CIPN and potential to monitor neuropathy progression.

## Teaser

Chemotherapy-induced peripheral neuropathy (CIPN) is a common dose-limiting toxicity with significant sequelae. There is a lack of standardized objective and reliable assessment tools in CIPN. In vivo reflectance confocal microscopy (RCM) imaging offers a non-invasive method to identify peripheral neuropathy markers, namely Meissner’s corpuscles. This article reports on the feasibility and value of RCM in CIPN.

**Implications for Practice**

There is no gold standard for diagnosis or assessment of chemotherapy-induced peripheral neuropathy (CIPN). In vivo reflectance confocal microscopy presents an objective, non-invasive approach to evaluate Meissner’s corpuscle density, a marker of peripheral neuropathy. Key findings from this pilot study suggest encouraging potential for the clinical utility of this tool in CIPN. Prospective assessment prior to chemotherapy demonstrated detection of subclinical deficits which may enable stratification of patients at risk. As the first study to demonstrate the feasibility of serial monitoring (during, post-treatment), this could elucidate patterns in the trajectory of CIPN (denervation/reinnervation) and guide the development of targeted therapies.

## Introduction

Chemotherapy-induced peripheral neuropathy (CIPN) is a major dose-limiting toxicity associated with many anti-cancer agents including platinums, taxanes, vinca alkaloids, and proteasome inhibitors affecting between 40% and 90% of patients depending on the regimen and assessment method, often forcing early cessation of treatment which may have implications for survival.^1-4^ Patients complain of paraesthesia (numbness), dysesthesia (tingling), and pain, especially in the glabrous surfaces of the hands and feet that can persist long after treatment, negatively impacting quality of life and return to productivity in cancer survivors.^3^

The threat posed by CIPN creates “clinical tensions”^5^ for patients and practitioners wherein a balance must be struck between aggressiveness of therapy to treat the cancer while still optimizing patient function and quality of life. Exacerbating this tension is the lack of consensus on optimal objective measures for identifying and characterizing CIPN.^6,7^ This also creates a challenge in the evaluation of novel treatments for CIPN prevention or reversal.^1,8^

Objective measures to assess peripheral neuropathy could be illuminating for CIPN. Degeneration of peripheral nerve endings often occurs early in response to injury as revealed using skin biopsy, which the European Federation of Neurological Societies acknowledges as a reliable and efficient diagnostic tool for small fiber neuropathies.^9-12^ Skin biopsy for epidermal nerve fiber (ENF) density assessment provides earlier detection of morphological and quantitative changes in peripheral nerves than neurophysiological tests (nerve conduction and nerve biopsy).^13^ It has proven useful in serial monitoring of progression and recovery in neuropathy due to diabetes, HIV, and idiopathic disease^9,10^; and was shown in patients with chronic CIPN, post-oxaliplatin or docetaxel, to yield a higher diagnostic sensitivity than nerve conduction studies.^14^ Disadvantages of skin biopsy include the risk of infection and failed wound healing^15^; and the use in glabrous skin is painful. The same site cannot be evaluated over time limiting generalized use as a tool in prospective studies of CIPN.

While nerve conduction studies can detect large (Aβ) nerve fiber deficits, and biopsy, small (Aδ, C) nerve fiber deficits, clinical characterization research in CIPN has revealed loss of function in both large and small fibres.^16^ This is reinforced by a recent systematic review of Quantitative Sensory Testing in CIPN studies demonstrating dysfunction in myelinated and unmyelinated sensory fibers across various types of chemotherapy.^17^ Quantitative Sensory Testing (QST) is shown to be a useful tool in the assessment of neuropathy^18,19^; however, the protocol can be time-consuming in clinical practice and is not entirely objective.

Meissner’s corpuscles (MCs) are another potential biomarker of peripheral neuropathy.^20^ Meissner’s corpuscle density at the fingertip was shown as a sensitive measure of diabetic neuropathy that correlated to ENF density.^21^ Meissner’s corpuscles, the most common low-threshold mechanoreceptors in glabrous skin, are innervated by both Aβ and C fiber axons and show rapidly adapting responses to low-frequency vibration, light touch, and microgeometric surface features. Meissner’s corpuscles may also encode some nociceptive stimuli.^22-24^ Structurally, MCs are ovoid encapsulated nerve terminals located within dermal papillae at the dermal–epidermal junction (DEJ) of fingers, palms, and soles of the feet.^22^ While 40% of sensory innervation in the hand is attributed to MCs, the highest density is distributed in the fingertips, with a decreasing trend proximally.^22,25^ Substantial MC reductions occur in HIV, diabetic, and hereditary peripheral neuropathies.^22^ Diminished density and sensory deficit are correlated to Parkinson’s disease severity.^26^ A case study of bortezomib-induced CIPN showed a parallel decrease in ENF and MC density by skin biopsy^27^; with similar findings reported in patients with vincristine and paclitaxel CIPN compared with healthy controls.^28^ Abnormalities in size, structure, and thinning or loss of myelin in MCs are associated with decreased tactile sensitivity.^22,23^

Importantly, MC density can be assessed non-invasively using in vivo reflectance confocal microscopy (RCM). Reflectance confocal microscopy images the cellular morphology of the epidermis and superficial dermis; with clear visualization of MCs, congruent to conventional histology for structures in the uppermost stratum corneum down to the reticular dermis^21,29,30^ and has been used successfully in various types of neuropathy^31-33^ and patients with multiple myeloma prior to chemotherapy.^34^ As RCM is non-invasive and an image site in glabrous skin can be indexed in reference to fingerprint whorls, it is a promising tool with the potential for monitoring dynamic changes in skin innervation over time.^21,29^

The goal of this pilot study was to use RCM to: (1) explore the range and distribution of MC density by age and gender in chemotherapy naïve, healthy controls; (2) investigate the correlation between MC density and validated measures of sensory and sensorimotor function; (3) compare baseline MC density of patients prior to receiving neurotoxic chemotherapy with age and gender-matched healthy controls; and (4) assess the feasibility of serial monitoring in patients receiving chemotherapy to explore changes in MC density over time and the relationship with patient-reported outcome measures and sensory/sensorimotor measures.

## Materials and Methods

### Study Design and Participants

As part of a prospective, multi-center observational study in CIPN, patients with cancer due to receive neurotoxic chemotherapy were recruited from the Lothian Regional Cancer Service/NHS Fife (ethical approval, REC 09/S1103/43). Written informed consent was obtained from all participants. Inclusion criteria consisted of: aged ≥18 years, receiving platinum agents (oxaliplatin, carboplatin), and/or taxanes (paclitaxel) as adjuvant treatment with curative intent of colorectal or gynecological cancer, with no previous history of neurological conditions, diabetes, alcohol excess, or pre-existing chronic pain/neuropathic conditions.

Healthy volunteers with no previous history of cancer, chemotherapy, diabetes, or neuropathy/neuropathic pain were recruited to investigate the normative range of MC density and as a control group to facilitate age and gender-matching with patients on the study.

### Experimental Paradigm

Patients were assessed at baseline prior to initiating chemotherapy, during chemotherapy (before cycles) and post-treatment (3 and 6 months). Healthy controls were assessed at one time point. The paradigm consisted of the following.

### In Vivo Reflectance Confocal Microscopy

#### Imaging Procedure

In vivo RCM (VivaScope 1500, Caliber I.D., Rochester, NY) was performed on the fingertip of digit V (palmar surface, glabrous skin) to visualize MCs. This site was chosen due to: the distribution of CIPN in the upper limb, a thinner epidermal layer on digit V, easily accessible, and avoidance of potential confounding of carpel tunnel symptoms considering the nerve supply of the hand in keeping with previous studies.^21,31-33^ Imaging was completed on the right hand of all participants and by one person, to ensure uniformity and consistency. Invasive skin biopsy was not conducted for comparison with RCM due to the potential risk of infection and compromised wound healing during chemotherapy (see Supplementary Material).

#### Meissner’s Corpuscle Identification and Quantification

As gray-scale RCM mosaics rely on “endogenous reflectance” for contrast, structural components of the skin such as melanin, organelles, and fibers appear bright (white) due to high refractive indices compared with surroundings.^35^ Meissner’s corpuscle identification coincided with the profile elucidated in previous studies.^21,31-33^ Meissner’s corpuscles typically located in the dermal papillae, if present, appeared as bright, ovoid structures (40-75 µm wide; ~150 µm in length).^36^ Distinguishing features included an encapsulated, heterogeneous appearance attributed to the internal lobulated structure of MCs, larger than inflammatory cells, and static during real-time imaging as opposed to blood flow in capillary loops. The absence of MCs was characterized by the empty “black pit” appearance in the dermal papillae.^21^

Meissner’s corpuscles were identified, traced through each successive depth of the mosaics (to avoid duplication) and counted manually by quadrant and tallied for each mosaic. Mosaics were reviewed by an experienced in vivo RCM microscopist, and MC quantification was conducted using the same, blinded approach. Where discrepancies occurred between reviewers, consensus was completed. Adobe Photoshop CC (2015) was chosen to calibrate image contrast, brightness and tabulate MC totals using the counting tool. As multiple mosaics were obtained for each control and patient, the mosaic image with the maximum number of MCs were included. Meissner’s corpuscle density was expressed as MC count per 3 × 3 mm mosaic.

### Quantitative Sensory Testing

Quantitative Sensory Testing is a standardized sensory exam using calibrated stimuli. It is a non-invasive, well validated, battery of mechanical and thermal tests to assess the function and performance of large (Aβ) and small (Aδ, C) nerve fibers along with their corresponding central pathways.^37,38^ The ability to phenotype somatosensory profiles can help identify pre-symptomatic changes and potential mechanisms for CIPN.^38,39^ The following parameters were assessed in the right upper limb (fingertip and thenar eminence) and lower limb: mechanical detection and pain thresholds, warm and cold detection and heat and cold pain thresholds as per previous research protocols (Supplementary Material).^27,39,40^

### Grooved Pegboard Test

Healthy controls and patients were asked to complete this task to assess manual dexterity and sensorimotor function (SupplementaryMaterial).^28^

### Patient-Reported Outcome Measures

To explore the relationship between MC density and PROMS, validated questionnaires were used to capture CIPN and symptom burden at each time point. The European Organisation for the Treatment of Cancer Quality of Life Questionnaire-Chemotherapy-induced peripheral neuropathy (EORTC QLQ-CIPN20) is designed to collect information regarding CIPN symptoms and functional limitations demonstrating good reliability, validity in detecting CIPN and responsiveness to change over time.^41^ It includes 20 questions divided into 3 subscales (sensory, motor, and autonomic) with higher scores indicating increasing severity. The Brief Pain Inventory (BPI) short form was used to assess pain intensity and interference/impairment.^42^ BPI scores are shown to be clinically relevant in patients with cancer.^43^ To characterize sensory disturbance/pain in the hands/feet, patients completed the 20 Word descriptors, a validated questionnaire consisting of commonly used terms for describing neuropathic pain (Supplementary Material).^27,44-46^

### Statistical Analysis

Descriptive statistics were presented where appropriate as mean ± SD and graphical illustration was used to highlight trends. Data from healthy controls were assessed for normality in distribution using histograms. Independent sample *t*-tests were performed to evaluate differences between gender while analysis of variance was conducted to investigate and stratify age-related changes by decade. Meissner’s corpuscle density and associations with QST, the Grooved pegboard test and PROMS were evaluated using Spearman’s correlation coefficients. Using the paired-sample *t*-test, patients’ baseline results were compared with age and gender-matched healthy controls. A repeated-measures analysis was adopted to model assessment of MC density from the same patient over time and address longitudinal measurements and potential changes from baseline, during chemotherapy cycles and post-treatment, while attempting to remove within-patient variability. Least squares means were deemed the most appropriate method to account for variability in cycles and follow up between patients. Intraclass correlation coefficient was calculated as a measure of inter-rater reliability between image reviewers. A *P*-value of <.05 was defined as statistically significant. As the main outcome was feasibility and acceptability of longitudinal measurements, the secondary outcomes comparing MC density between cancer patients and healthy controls, and the data over time were collected and analyzed to inform a future larger scale trial. Therefore, no adjustments for multiple testing were performed.

## Results

### Demographics

Forty-five healthy controls, mean age 58.9 ± 12.1 years (range 24-81) and 9 patients with cancer receiving neurotoxic chemotherapy, ranging in age from 54 to 72 years were evaluated (Table 1).

**Table 1.** Demographics of study group (patients and healthy controls).

Demographics	Age and number of participants
Healthy controls	
Age, mean (SD)	58.9 (12.1) years
Gender	
Male	20
Female	25
Patients	
Age, mean (SD)	63.9 (8.2) years
Gender	
Male	5
Female	4

### Meissner’s Corpuscle Density in Healthy Controls

**Figure 1.**
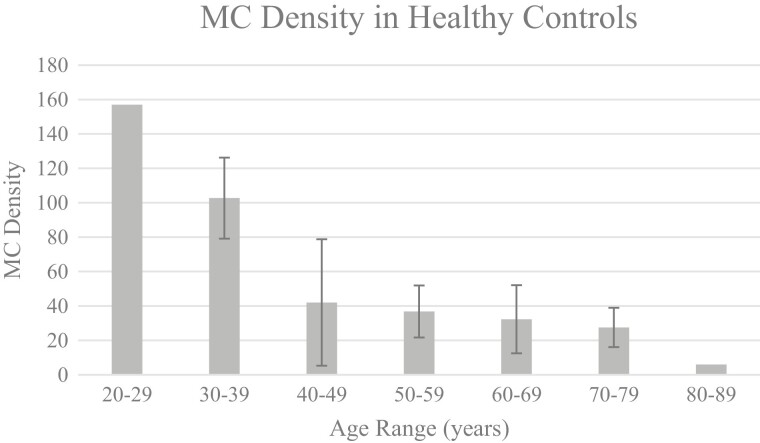
MC density (mean) in healthy controls across age range. This figure highlights the inverse relationship between MC density at the fingertip and age in healthy controls. Meissner’s corpuscle density was expressed as the maximum MC count per 3 × 3 mm mosaic. Error bars represent mean ± standard deviation (standard deviations were not provided as there were too few patients in ages 20-29 and 80-89 years). Analysis of variance by decade was statistically significant (*P < .*0001).

Analysis of MCs revealed statistically significant differences by gender with females exhibiting a greater mean density at the fingertip compared with males (49.88 ± 7.12 MC/3 × 3 mm vs. 30.9 ± 4.18 MC/3 × 3 mm; *P = .*028). Significant variances were also observed across age range (*P < .*0001) (Fig. 1).

### Correlation with Validated Sensory and Sensorimotor Measures

#### Quantitative Sensory Testing Parameters

Meissner’s corpuscle density at the fingertip was negatively correlated with mechanical detection threshold at both the fingertip (*ρ* = −0.507; *P = .*001) and lower limb (*ρ* = −0.463; *P = .*006). Associations were also observed between MC density and thermal thresholds in the hand. Meissner’s corpuscle density at the fingertip was negatively correlated with warm detection threshold (*ρ* = −0.471; *P = .*003) and in contrast positively correlated with cold pain detection threshold (*ρ* = 0.492; *P = .*002). No significant correlations were apparent between MC density and mechanical pain or thermal thresholds in the lower limb (Table 2).

**Table 2.** Correlations between MC density and validated measures (QST and Grooved pegboard test).

			MDT upper limb	MDT lower limb	WDT upper limb	CPT upper limb	PegboardDominant hand	Pegboard non-dominant hand
Spearman’s rho	MC density consensus count	Correlation coefficient	−0.507**	−0.463**	−0.471**	0.492**	−0.378*	−0.447**
		Sig. (2-tailed)	*P = .*001	*P = .*006	*P = .*003	*P = .*002	P = .021	*P = .*006

*P < .05; **P < .01.

Abbreviations: MC, Meissner’s corpuscles density; QST, Quantitative Sensory Testing; MDT, mechanical detection threshold; WDT, warm detection threshold; CPT, cold pain threshold.

#### The Grooved Pegboard Test

Completion of the Grooved pegboard test in seconds was negatively correlated with MC density (fingertip) in the dominant (*ρ* = −0.378; *P = .*021) and non-dominant hand (*ρ* = −0.447; *P = .*006; Table 2).

#### Meissner’s Corpuscle Density: Patients’ Baseline vs Healthy Controls

**Figure 2.**
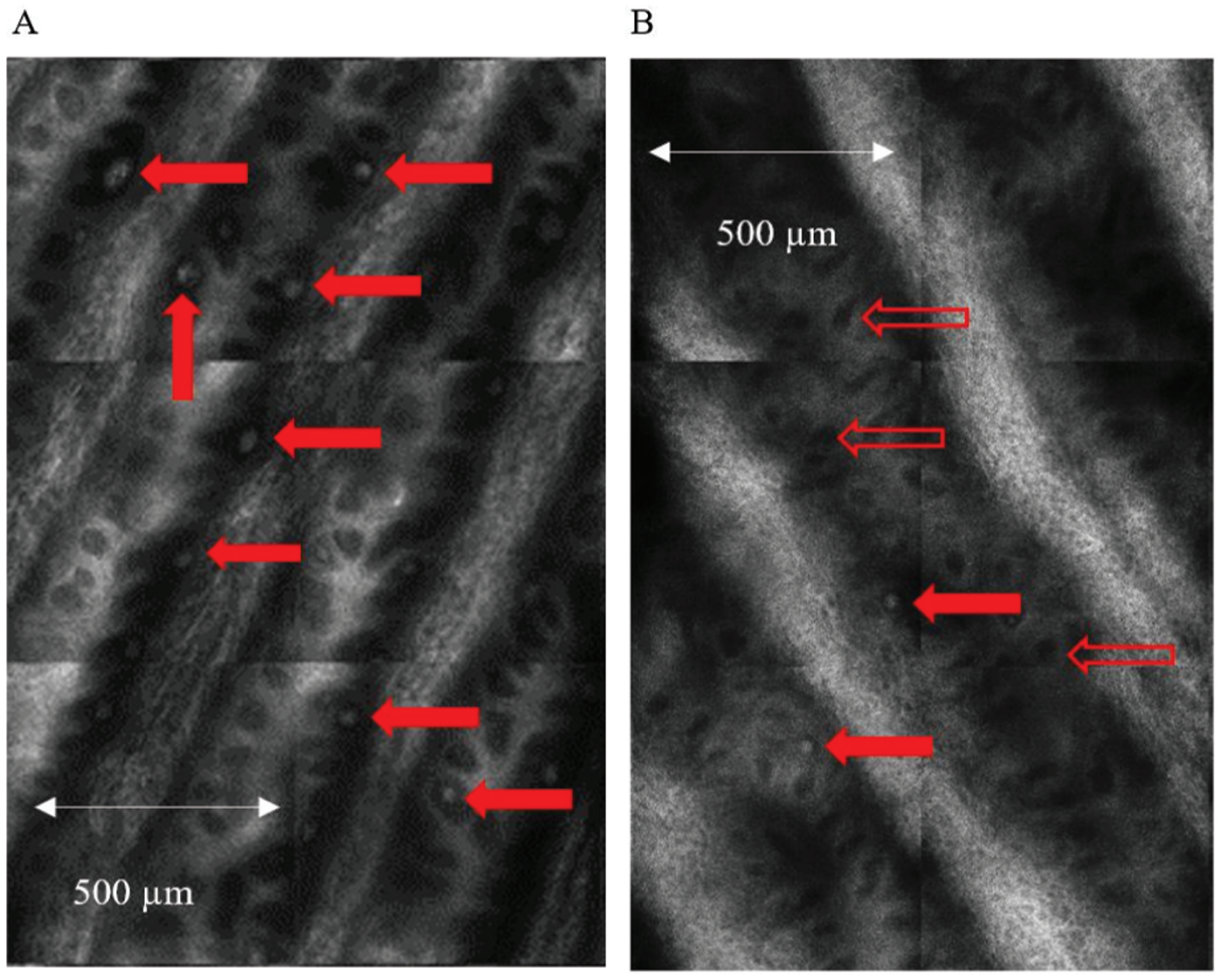
In vivo reflectance confocal images of Meissner’s corpuscles. Enlarged view of quadrants from an “optical biopsy” mosaic obtained from the fingertip (digit V) of (**A**) healthy control and (**B**) patient at baseline prior to treatment. Meissner’s corpuscles (MCs) which appear as bright, ovoid white structures are normally located in the dermal papillae and are indicated by the solid red arrows. The open red arrows show empty dermal papillae which appear as dark regions due to the absence of MCs. The comparison highlights the difference in MC density between the two groups.

Comparison between the groups revealed a higher MC density in healthy controls, 35.44 ± 14.11 MC/3 × 3 mm vs 22.00 ± 9.29 MC/3 × 3 mm (*P = .*026) in patients with cancer imaged at baseline, prior to chemotherapy (Fig. 2), mirroring deficits in cold detection threshold and the Grooved pegboard test (*P = .*050).

### Assessing MC Density Over Time

Patients were imaged for a total of 34 assessments. From baseline, a decreasing trend in mean MC density was noted to cycle 4 with an increase in cycle 5/6, decreasing in cycle 7 with further decline in the post-treatment follow-up period. Changes in mean MC density were statistically significant (*P < .*001) except for cycle 8 (*P = .*072). Due to the sample size and data set, *P*-values should be interpreted cautiously (Table 3). Examination of individual patients revealed some variability in MC density trend over time (Supplementary Fig. S1).

**Table 3.** Mean MC density over time in patients (repeated-measures analysis).

Time point	MC density estimate ± SEM	*P*-value
B	22.00 ± 2.92	*<.*0001
C2	18.37 ± 5.32	.0006
C3	17.32 ± 2.61	*<.*0001
C4	8.94 ± 1.88	*<.*0001
C5	42.52 ± 3.41	*<.*0001
C6	38.29 ± 4.24	*<.*0001
C7	18.86 ± 1.15	*<.*0001
C8	25.62 ± 14.25	.0722
3M	18.73 ± 3.35	*<.*0001
6M	11.94 ± 1.88	*<.*0001

Baseline (B) prior to starting chemotherapy; chemotherapy cycles (C2-C8); post-treatment follow-up 3 months (3M) and 6 months (6M); SEM, standard error of the mean.

Statistically significant correlations across timepoints were noted between MC density and PROMS specifically: EORTC QLQ-CIPN20 motor (*ρ* = −0.432; *P = .*011) and autonomic subscales (*ρ* = −0.408; *P = .*017) and the frequency of “cold” on the 20 Word descriptors (*ρ* = −0.404; *P = .*018). No associations of significance were observed with EORTC QLQ-CIPN20 sensory subscale or BPI (Supplementary Table S1).

Meissner’s corpuscle density was negatively correlated with QST: warm detection (ρ=-0.356; *P = .*039) and cold pain (*ρ* = −0.489; *P = .*003) thresholds. On the Grooved pegboard test (non-dominant hand), a borderline association was observed (*ρ* = −0.332; *P = .*05; Table 4 and Supplementary Figs. S2-S5).

**Table 4.** Summary of significant correlations between MC density and validated measures over time (QST, grooved pegboard test, PROMS).

			WDT upper limb	CPT upper limb	Pegboard dominant hand	EORTC QLQ-CIPN20 motor subscale	EORTC QLQ-CIPN20 autonomic subscale	20 word descriptors-cold
Spearman’s rho	MC density consensus count	Correlation coefficient	−0.356*	0.489**	−0.332	−0.432*	−0.408*	−0.404*
		Sig. (2-tailed)	*P = .*039	*P = .*003	*P = .*05	*P = .*011	*P = .*017	*P = .*018

*P < .05; **P < .01.

Abbreviations: MC, Meissner’s corpuscles density; QST, Quantitative Sensory Testing; WDT, warm detection threshold; CPT, cold pain threshold.

### Intraclass Correlation

**Figure 3.**
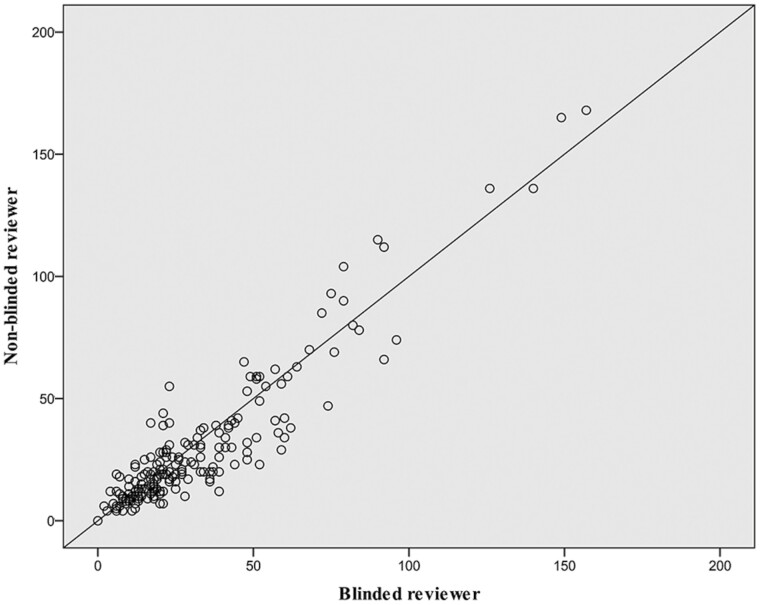
Inter-rater reliability of MC density count between image reviewers. On this scatterplot, circles represent the comparison of Meissner’s corpuscle (MC) density count between the blinded reviewer (C.A.-F) and non-blinded reviewer (S.R.R.) for the multiple optical biopsies obtained for healthy controls and patients. Of the 229 images reviewed, the intraclass correlation was 0.961 (95%CI: 0.946-0.971; *P* < .0001).

Inter-rater reliability of MC densities was investigated between 2 reviewers, a clinical research fellow (S.R.R.) and an experienced RCM microscopist (C.A.-F.). Intraclass correlation was calculated for the multiple mosaics from controls and patients. Of the 229 images reviewed, the intraclass correlation was 0.961 (95%CI: 0.946-0.971; *P < .*0001; Fig. 3).

## Discussion

The main findings show that RCM revealed an age-related decrease in MC density in healthy controls that parallels previous findings using traditional skin biopsy histological methods. Clear gender dimorphism in MC density was also observed. Importantly, MC density showed strong correlations to QST function as would be predicted based on the modalities of sensory information encoded by MCs, and as well as to sensory modalities encoded by ENFs, suggesting that MC density assessed by RCM provides an indirect measure of ENF density. The practicability of re-imaging MC density longitudinally demonstrated significant relationships with QST and PROMS. Overall, there was strong/high inter-rater reliability in MC density assessment.

## Normative RCM Measures of MC Density Correlate to QST Measures

Data from healthy controls (24-81 years) revealed that MC density was inversely related to age and varied substantially across the 6 decades. Our findings parallel previously established skin biopsy studies where MC density was shown as highest in young children, decreasing in the second and third decades with continual reductions estimated at 50% by the eighth decade.^23,47^ This is confirmed by additional biopsy studies although high variability among individuals contributed to slightly different patterns of decline.^36,48^ Meissner’s corpuscles are rapidly adaptive with small receptive fields providing “decisive sensory input” for object size, shape, texture, location, and 2-point discrimination.^25,36^ Our finding of a statistically significant negative correlation between MC density and mechanical detection threshold at the fingertip is in keeping with this physiological role.^36^ Similarly, a negative association with grooved pegboard performance was predictable. The relationship between reduced MC density and age dovetails with the reduction in tactile acuity with age.^36,49^ We also found that females exhibited greater MC density than males consistent with previous RCM^21^ and skin biopsy studies^50^ as well as our QST findings. The gender effect may be a function of finger volume whereby smaller finger size in females results in finer tactile acuity.^36,47,51^ While this may translate to a “finer-grained” afferent neural image, consideration should also be given to a thicker stratum corneum predominantly found in males.^51,52^

The MC density results on RCM are also of interest in relation to ENF density. Meissner’s corpuscles are multiafferented, containing myelinated, and unmyelinated primary afferent nerve endings.^21,24^ Associations between MC density at the fingertip and mechanical detection in the lower limb suggest that relative density at isolated testing sites yields insight to generalized systemic innervation density. This is supported by a study of patients with sensory neuropathy who exhibited a reduction in ENF density in both the upper and lower limbs.^53^ As C-fibers transduce innocuous warm sensations and noxious thermal stimuli this may account for the negative and positive correlations noted with thermal thresholds, warm detection, and cold pain, respectively, in our study.^54^ This is relevant as QST studies have delineated a connection between elevated warm threshold and skin denervation in small fiber neuropathy.^10^ Epidermal nerve fiber density shows the same association to age as for MCs shown here. In one study, the highest ENF densities were detected in the youngest group (10-19 years), while gender did not have significant effect.^8^ Conversely, in a homogenous Scandinavian population of 106 healthy controls (ages 16-92) ENF density decreased significantly with increasing age and in males compared with females.^55^ Higher ENF densities in females may underlie the increased sensitivity to thermal and pain parameters exhibited on QST.^39^

## Comparison of Baseline MC Density in Patients with Healthy Controls

In this study, patients with colorectal and gynecological cancer had significantly lower MC density at baseline prior to receiving neurotoxic chemotherapy compared to age and gender-matched healthy controls. Similarly, an RCM study of 12 chemotherapy-naïve patients with multiple myeloma demonstrated decreased MC density at baseline and nerve fibre deficits (Aβ, Aδ, and C) compared with healthy controls^34^; which dovetails to the finding of subclinical pre-treatment neuropathy in patients with colorectal cancer,^40^ and reduced corneal nerve fiber density in patients with gastrointestinal cancer prior to chemotherapy.^56^ This mirrors results from a mixed cohort of patients with pre-treatment cancer where large and small fiber sensory dysfunction (39.7% and 50%, respectively) was detected in the fingers and/or toes using QST.^57^ More generally, studies in HIV,^31^ Charcot-Marie-Tooth,^32^ and diabetic neuropathy,^33^ have shown meaningful differences in MC density between patients and controls with RCM. In HIV+ patients, MC density in digit V coincided with signs of sensory peripheral neuropathy in patients with and without overt signs and is significant, considering nerve conduction of the ulnar, medial plantar and sural nerves revealed comparable findings to controls.^31^ This suggests that RCM may detect subclinical signs earlier in asymptomatic patients.^31^ Correspondingly, skin biopsy is considered more sensitive in identifying small nerve fiber neuropathy vs nerve conduction studies addressing large fiber function.^9^ In a study of patients with cancer nerve fiber density was found to be markedly reduced prior to commencing chemotherapy (platinum or taxanes).^58^ These results reinforce similarities in the potential utility of RCM in peripheral neuropathy to the role and capacity of skin biopsy. Deficits in these components may highlight patients at risk of sensory loss as seen in diabetic neuropathy.^37^

## Prospective Exploration of MC Density in Patients Over Time

Adopting a novel approach to investigate the viability of re-imaging patients longitudinally yielded preliminary yet interesting findings. Assessment of MC density from baseline, revealed a significantly decreasing trend during most cycles with increasing fluctuations in the mid-late cycles and persistent reduction post-treatment. Importantly, at 6 months follow up, MC density did not revert to baseline which was shown to be substantially lower compared with age and gender-matched healthy controls. Although RCM has not been utilized for repeated measures in this demographic, the results parallel a similarly conducted prospective skin biopsy study in 8 patients with colorectal cancer, where a significant reduction in ENF density was observed at baseline and during chemotherapy, with progression continuing post-treatment suggestive of axon loss (sensory and motor).^59^ Axonopathy and peripheral nerve degeneration are postulated to be fundamental in the development of CIPN^60^ and may explain the coasting phenomenon at 6 months reported by Burakgazi et al. and mirrored in our study.

The trajectory of changes in MC density using RCM in the context of potential underlying mechanisms may provide insight into the capacity of this tool in CIPN. Reductions in MC density from baseline to as early as cycle 2 with persistence until cycle 4 may concur with the “dying-back”^61^ process where the distal nerve endings are susceptible and predominantly affected by neurotoxic chemotherapy, characteristic of a length-dependent neuropathy.^3,7^ This coincides with findings in patients with sensory neuropathy, where decreased MC density on histology equated to skin biopsy, a sensitive indicator of early reductions in ENF density.^21,62^ Biopsy results from patients with cancer also exhibit this pattern,^58^ suggesting that subclinical deficits are amplified when chemotherapy is commenced, potentially influencing CIPN development.

While the increasing trend in MC density in cycles 5/6 seem paradoxical compared with earlier reductions, they may reflect a denervation–reinnervation paradigm. Animal model studies and skin biopsy in humans have confirmed that following denervation, MCs maintain a functional ability for reinnervation.^21^ Comparatively, epidermal nerve fibers demonstrate a similar capacity.^10,13^ In a prospective study of 12 patients with cancer (colorectal, prostate, and breast), Koskinen et al. noted increases, decreases, and even normalization of intraepidermal nerve fiber (IENF) density prior to and during chemotherapy corresponding with the findings in our study.^58^ While axonal regeneration and collateral sprouting are implicated in the reinnervation process,^63,64^ in patients with painful sensory neuropathy, greater branching, and sprouting of IENFs have been suggested as an early marker of nerve fiber dysfunction.^10,64^ Another consideration is that the patients assessed at baseline using RCM varied in age (54-78 years). Given the differences observed by age in healthy controls in Fig. 1, greater MC densities (Supplementary Fig. S1) may be more reflective of “normalization.” Sampling/selection bias may have played a role. Although preliminary, the differing “evolutional patterns” seen in this study are consistent with previous longitudinal descriptions of ENF density in patients with cancer.^58,59^

## Meissner’s Corpuscles Density and Longitudinal Correlations

The adverse implications of CIPN on activities of daily living and functional outcomes are well documented. The paradigm in this study provided a novel approach to interrogate some of these relationships over time using validated objective and subjective measures. The association observed between MC density and thermal thresholds (warm detection and cold pain) on QST are suggestive of small nerve fiber dysfunction. These findings are meaningful given that small fiber damage is a prerequisite for painful neuropathy. The link between nociception and pathogenesis of neuropathic pain coincides with data from animal models and clinical studies involving assessment of IENF density.^65,66^ Unlike the observations in healthy controls in this study, no significant correlation was discernible between MC density and mechanical detection threshold (MDT) in patients. Despite the predominance of small fiber dysfunction, there is suggestion of large fiber impairment based on an increasing trend in MDT over time (Supplementary Fig. S2) and a borderline correlation between MC density and the Grooved pegboard test in the dominant hand (*ρ* = −0.33; *P = .*05). This may reflect the differing patterns of nerve fiber involvement consistent with previous research. In a prospective multiple myeloma study, Richardson et al. found that in patients with peripheral neuropathy at baseline the majority presented with pure small fiber involvement while following chemotherapy patients exhibited both pure small fiber and a combination of large and small inovement.^62^

Significant relationships were also apparent between MC density and PROMS. Scores on the motor and autonomic subscales on the EORTC QLQ-CIPN20 questionnaire suggest that reduced MC density is associated with increasing symptom burden. Although the correlation with the EORTC QLQ-CIPN20 sensory subscale and total score did not achieve statistical significance, an increasing trend was observed over time (Supplementary Figs. S3 and S4). The findings are concordant with CIPN symptom clusters, specifically the motor-sensory neuropathy cluster characterized, for example, by “difficulty manipulating small objects” (coinciding with the Grooved pegboard results).^67^ The 20 word descriptors was useful in qualifying symptoms not captured on other PROMS (Supplementary Fig. S5). The prevalence of the “cold” descriptor and correlation with MC density is likely due to the larger proportion of patients with colorectal cancer treated with oxaliplatin in this cohort. The BPI results are as expected as many patients have difficulty defining their symptoms as solely painful.

As the first study to explore how MC density correlates with QST and PROMS over time several factors need to be taken into consideration when interpreting the results. Small sample size, mixed cohort (differences in type of cancer, chemotherapy, and number of cycles), and variability in data across timepoints may all play a role. Importantly, patients on this study were assessed prospectively, multiple times in comparison to previous cross-sectional RCM studies of patients with established neuropathy. While preliminary, these results allude to the complex, heterogeneity of clinical manifestations in CIPN and highlight the multifactorial nature of the relationships.

## Feasibility of Use in CIPN

Repeated measurements facilitated insight into the practical and technical capabilities of RCM. Advantages included: user-friendly software for capturing images, maneuverability, and ergonomic positioning of the microscope head to accommodate patients’ comfort. Patient feedback indicated that imaging was well tolerated, painless, and relatively quick. Variability in thickness of the stratum corneum (possibly attributed to occupation/hobbies) required a greater depth to reach the DEJ. A limitation of RCM is the maximum depth of penetration (350-400 µm) on acral surfaces, compromising image quality (loss of resolution and contrast).^35^ While patient movement may result in misaligned images, this is minimized by re-imaging. Training in image acquisition and interpretation is required: It is possible for non-specialist staff to be trained in using this technique quantitatively, with use of guidelines^68^ reported to increase accuracy. It is a technique where there is the potential to use artificial intelligence approaches to interpretation in the future.^69^

Real-time imaging in conjunction with PROMS facilitated a unique forum to engage, educate, and discuss CIPN while patients’ active participation epitomized a patient-centered, shared model of care. Research supports the critical concept that patients are eager to gain more information regarding toxicities when considering treatment regimens.^7,70^

## Overall Limitations

The sample size for the patients was considerably smaller than the cohort of healthy controls (45) which may have implications in interpretation of the results. However, these data can be used to inform sample size calculations for future studies. Manual counting of MCs was time-consuming and required additional software for image optimization. Advances in automated delineation of the DEJ may assist with developing RCM algorithms for automated qualification, recognition, and quantification of MC density.^71^

## Conclusion

The findings from this pilot study support the clinical utility of RCM in CIPN. Data from healthy controls represent a key step toward generating normative reference ranges, revealing that RCM captures sensory information encoded by MCs and ENF density while correlated with validated measures of sensory and sensorimotor function. This together with the ability to identify subclinical deficits in patients prior to chemotherapy may serve as a benchmark for future studies to expand on with implications for risk stratification of CIPN.

There is a need for establishing “real-time” clinically meaningful changes in patient-reported outcomes^72^ as well as developing reliable clinical assessments due to the lack of gold standard in CIPN.^73^ Integrating PROMS with QST, the Grooved pegboard test and repeated assessment of MC density facilitated a window for direct correlation of nerve fiber function and individual anatomical/structural changes in conjunction with patients’ experience of symptoms. This multimodal clinical characterization provides insight into mechanistic underpinnings. The consistent relationship between MC density and thermal thresholds suggests responsiveness to change over time and the capacity to capture small nerve fiber dysfunction, a potential predictive biomarker of painful neuropathy. Building on this framework, further longitudinal studies in larger cohorts are required, with additional investigation for determining sensitivity and specificity. This non-invasive, painless, objective method for serial monitoring in real-time and individualized mechanism-based approach of phenotype profiling may aid the development of therapeutic and preventive interventions^74^ and assist with personalized treatment.

## Supplementary Material

oyac106_suppl_Supplementary_Materials

oyac106_suppl_Supplementary_Methods

## Data Availability

The data underlying this article are available in the article and in its online supplementary material.
