# Supplementary material for: The Value of In Vivo Reflectance Confocal Microscopy as an Assessment Tool in Chemotherapy-Induced Peripheral Neuropathy: A Pilot Study
Source: Oncologist. 2022 Jun 15;27(8):e671–80. doi: 10.1093/oncolo/oyac106 (PMC9355818; doi:10.1093/oncolo/oyac106)
Supplement: oyac106_suppl_Supplementary_Materials [file oyac106_suppl_supplementary_materials.docx]

Supplementary Results (Figures and Tables)

**Figure S1 MC Density (actual count) over Time from each Patient**

This figure demonstrates the change in Meissner’s corpuscle (MC) density in cancer patients over time as assessed at baseline before starting chemotherapy (B), prior to each chemotherapy cycle (C2-C8) and follow up post-chemotherapy at 3 months (3M) and 6 months (6M). The coloured symbols represent the actual MC density count from each patient (colorectal cancer patients 1, 3-6, 8,9; gynaecological cancer patients 2, 7). MC density was expressed as the maximum MC count per 3x3 mm optical biopsy. Note gaps in each patients’ trajectory represent data not available at that time point.

**Table S1 MC Density correlated with EORTC QLQ-CIPN20 questionnaire**

|  | | | EORTC QLQ-CIPN20  Sensory  subscale | EORTC QLQ-CIPN20  Motor  subscale | EORTC QLQ-CIPN20  Autonomic  subscale | EORTC QLQ-CIPN20 Total Score |
| --- | --- | --- | --- | --- | --- | --- |
| Spearman's rho | MC density consensus count | Correlation Coefficient | -.222 | -.432^*^ | -.408^*^ | -.272 |
|  |  | Sig. (2-tailed) | *p*= 0.901 | *p*= 0.011 | *p*= 0.017 | *p*= 0.119 |

MC (Meissner’s corpuscles) density


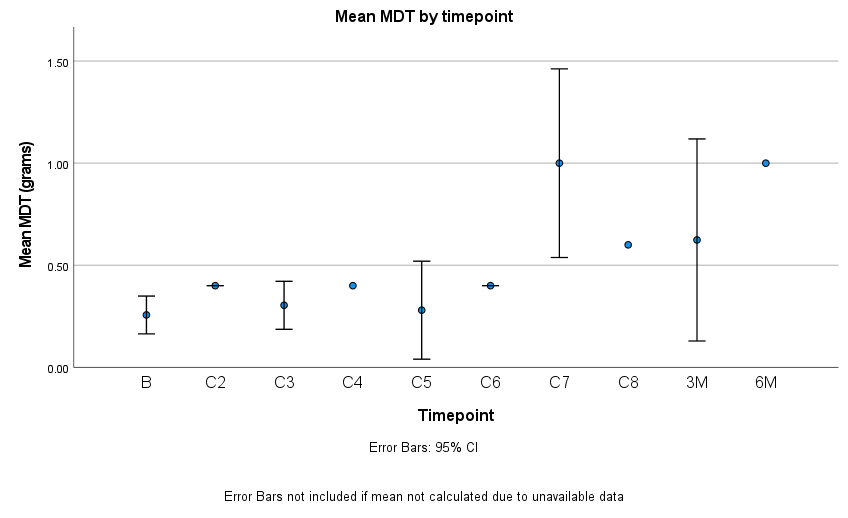


**Figure S2 Mean Mechanical detection threshold over time**

This figure demonstrates the change in mechanical detection threshold (MDT ±SEM) in cancer patients over time as assessed at baseline before starting chemotherapy (B), prior to each chemotherapy cycle (C2-C8) and follow up post-chemotherapy at 3 months (3M) and 6 months (6M).


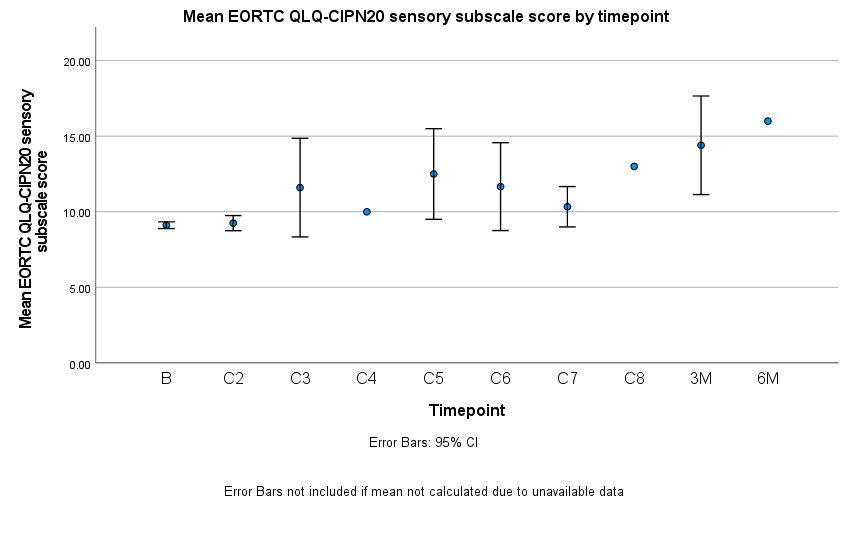


**Figure S3 Mean EORTC QLQ-CIPN20 sensory subscale score over time**

This figure demonstrates the change in EORTC QLQ-CIPN20 sensory subscale score (±SEM) over time in cancer patients as assessed at baseline before starting chemotherapy (B), prior to each chemotherapy cycle (C2-C8) and follow up post-chemotherapy at 3 months (3M) and 6 months (6M).


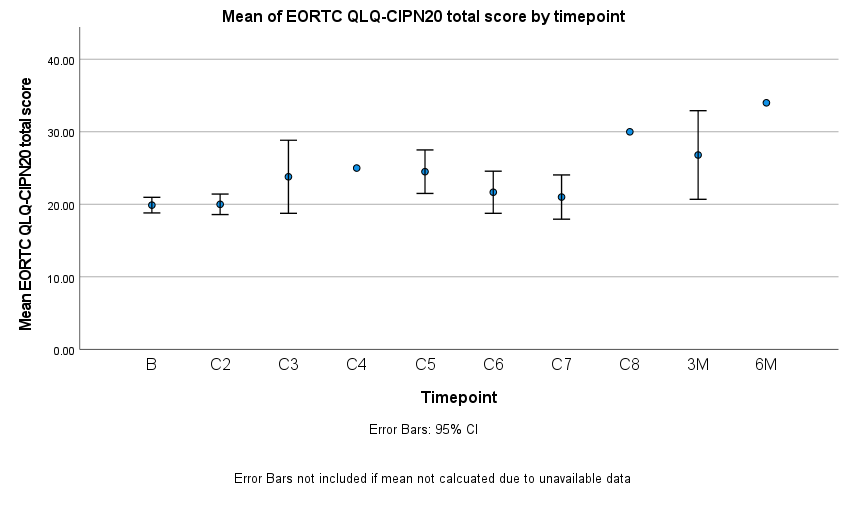


**Figure S4 Mean EORTC QLQ-CIPN20 total score over time**

This figure demonstrates the change in EORTC QLQ-CIPN20 total score (±SEM) over time in cancer patients as assessed at baseline before starting chemotherapy (B), prior to each chemotherapy cycle (C2-C8) and follow up post-chemotherapy at 3 months (3M) and 6 months (6M).

**Figure S5 Frequency of 20 Word Descriptors over time**

The graph depicts the frequency each word on the descriptor list was chosen by patients over time, during cycles and post-treatment follow up to describe symptoms in their hands and feet.
