# Supplementary material for: The Value of In Vivo Reflectance Confocal Microscopy as an Assessment Tool in Chemotherapy-Induced Peripheral Neuropathy: A Pilot Study
Source: Oncologist. 2022 Jun 15;27(8):e671–80. doi: 10.1093/oncolo/oyac106 (PMC9355818; doi:10.1093/oncolo/oyac106)
Supplement: oyac106_suppl_Supplementary_Methods [file oyac106_suppl_supplementary_methods.docx]

Supplementary Methodology

**In vivo reflectance confocal microscopy**

*Imaging procedure*

In brief, the principles and foundations of RCM are based on its components which include: a point source of light, condenser, objective lenses and a point detector.^35^ Using a laser as a light source (near-infrared wavelength), illumination is directed on a small area of skin. The light emanating from this focus plane is diverted through a pinhole which permits light only from this region towards the detector in order to generate thin horizontal sections.^29, 35^

The first step in each RCM procedure was to collect a macroscopic image of the fingertip as a reference point for follow-up scans using a handheld digital dermatoscope (VivaCam, MAVIG GmbH, Munich, Germany). The high magnification scan used the stratum corneum, or first layer of epidermis, as a point of reference from which to set the depth of imaging in the tissue. RCM images were viewed in real-time on a computer monitor (grayscale images), and a series of 0.5 x 0.5 mm images were captured and “stitched” together to create 3x3 mm mosaics in single, en face planes in the epidermis, dermal-epidermal junction (DEJ) and superficial papillary dermis. To detect the most superficial MCs, the first mosaic was captured in the suprabasal layer. Sequential, mosaic images at successive depths (20 µm) were obtained to capture the full range of MC visibility (due to the vertical nature of MC structure) therefore creating a non-invasive “optical biopsy.”^29, 35^ This process was repeated and multiple optical biopsies were taken at the fingertip to investigate the scope of MC distribution with a completion time of approximately 10-15 minutes. Healthy controls were assessed with RCM at a single time-point while patients were evaluated at baseline, during cycles (maximum 8) and post-treatment follow up (3 and 6 months).

**Quantitative Sensory Testing (QST)**

a) Mechanical detection threshold

This was measured using a standardised set of von Frey monofilaments (North Coast Touch-Test Sensory Evaluators) which exert bending forces between (0.008-300g) in order to assess Aβ fibre functions. In accordance with previous studies, an "up-down manner" with a series of ascending and descending stimuli intensities were applied to the sites until the same monofilament was detected on three applications. This was defined as the mechanical detection threshold.^27^

b) Mechanical pain threshold

A blunted 30-gauge needle attached to weights (8,10,16,20,32,64 or 128g) and placed in a syringe was applied to the sites in ascending order to assess Aδ fibre function. Participants were asked to determine when the stimulus felt sharp or painful. Three trials were conducted at each site and the mean was defined as the sharpness detection threshold.^27^

c) Thermal detection and pain thresholds

To evaluate sensory modalities such as warm and cold sensation detection along with heat and cold induced pain, the TSA-II NeuroSensory Analyzer (Medoc, Inc.), a computer-controlled device designed to assess Aδ and C fibre function was utilised. To conduct the assessment, a Peltier probe (3 cm x 3 cm) was applied to the skin of the test sites, the thenar eminence in the upper limb and 2 cm above the medial malleolus in the lower limb. The thermal testing algorithm was based on the method of limits which involved patients signalling by clicking a button when they first perceived a change in temperature (warm or cold detection) and when the stimuli felt painful (hot or cold) in effect halting the stimulus.^40^ For warm detection and heat pain, the baseline temperature was set to 32°C to maximum of 50°C. For cold detection and cold pain, the temperature again started at a baseline of 32°C and decreased to 0°C. For each of the four sensory modalities, 3 consecutive stimuli were generated and the mean was defined as the thermal detection (warm or cold) and pain (hot and cold) threshold.^39^

**Grooved pegboard test**

Patients were asked to place 25 pegs in a 5x5 slotted, grooved pegboard as quickly as possible in an ordered approach either by rows or columns. As the pegs contain a “key” which must be rotated prior to inserting in the pegboard, this process involved complex visual-motor coordination when compared to a non-grooved pegboard in order to assess manual dexterity and sensorimotor function. The task was completed first with the dominant hand and then the non-dominant hand (both trials were timed).^28^

**Patient-reported Outcome Measures (PROMS)**

The European Organisation for the Treatment of Cancer Quality of Life Questionnaire- Chemotherapy-induced peripheral neuropathy (EORTC QLQ-CIPN20) was designed to specifically address CIPN as an additional component to the European Organisation for the treatment of cancer quality of life questionnaire EORTC QLQ-30. The EORTC QLQ-CIPN20 questionnaire was developed to obtain information regarding symptoms and functional limitations as a result of CIPN in order to capture patients’ subjective experience over the past week in a self-reported manner. It is comprised of 20 questions which are divided into 3 subscales: sensory (9 questions), motor (8 questions) and autonomic (3 questions). The scores based on a 4-point Likert scale range from 1= “not at all, 2= “a little,” 3= “quite a bite,” to 4= “very much.” The sensory scores range from 9 (baseline) to 36, motor scores from 8 (baseline) to 32 and autonomic 3 (baseline for men) and 2 (baseline for women) to 12 with higher scores indicating a greater degree of symptom burden. A study by Lavoie et al. demonstrated the validity, reliability and responsiveness to change over time in assessing patients with CIPN.^41^

**20 word descriptors**

Previous CIPN studies conducted by MD Anderson Cancer Research Centre have included an “ideal” list of 20 word descriptors commonly used and validated for describing neuropathic pain.^27, 44-46^ The list includes the following: drilling, stabbing, sharp, squeezing, tugging, tearing, dull, splitting, hot, burning, cold, spreading, flashing, flickering, throbbing, itching, electric current, shooting, tingling and numb. Patients were asked to identify all applicable word descriptors from the above list of words to characterise sensory disturbance and or pain in their hands or feet.
